# Potential Renal Acid Load, Salivary Buffer Capacity and Healthy Eating Index as Predictors of Children’s Dental Caries: A Cross-Sectional Study

**DOI:** 10.7759/cureus.39513

**Published:** 2023-05-26

**Authors:** Elif Kibaroglu, Ayca Kurt, Yagmur Demirel Özbek, Ozlem Saral, Omer Hatipoglu

**Affiliations:** 1 Pediatric Dentistry, Recep Tayyip Erdogan University, Rize, TUR; 2 Dentistry Faculty, Recep Tayyip Erdogan University, Rize, TUR; 3 Nutrition and Diet, Recep Tayyip Erdogan University, Rize, TUR; 4 Health Sciences Faculty, Recep Tayyip Erdogan University, Rize, TUR; 5 Restorative Dentistry, Niğde Ömer Halisdemir University, Nigde, TUR

**Keywords:** healthy eating index, salivary buffer capacity, potential renal acid load, dental caries, child

## Abstract

Introduction: Dental caries is one of the most common childhood diseases. This study purposed to investigate the prediction capability of potential renal acid load (PRAL), salivary buffer capacity (SBC), and Healthy Eating Index (HEI) on children's dental caries.

Methods: The decay, missing, filing, and teeth for primary teeth (dmft)/Decay, Missing, Filling, and Teeth for permanent teeth (DMFT) indexes of the children aged 7-12 years who applied to our faculty were recorded. Approximately 1 mL of unstimulated saliva samples were collected, and SBC was evaluated. PRAL and HEI scores were calculated by entering the data in the form of a daily nutrition record of the children into the BeBiS software (Ebispro for Windows, Stuttgart, Germany). The association of dental caries indices with PRAL, SBC, and HEI was analyzed using an independent sample t-test. A binomial logistic regression analysis was performed to predict the dental caries burden. The statistical significance level was adjusted to a=0.05.

Results: A total of 150 children, 88 (58.6%) females and 62 (41.4%) males, were included in the study. Significant differences were found between the low and high dental caries groups for dmft regarding PRAL and SBC (p<0.001). A significant difference was found between the low and high dental caries groups for DMFT in terms of SBC (p<0.05).

Conclusion: In our study, established regression models significantly predicted dental caries in primary teeth. SBC was the most influential factor in predicting dental caries compared to PRAL and HEI. There was a significant relationship between SBC, PRAL, and caries in primary teeth. In the model we created, the strongest predictor was SBC.

## Introduction

Dental caries is a chronic, infectious, and multifactorial disease in which tooth structure is destroyed by acidic by-products produced by the fermentation of dietary carbohydrates [1,2]. Caries risk indicators are the variables that are assumed to directly cause or contribute to caries formation. The most commonly used caries risk indicators are low salivary flow rate, cariogenic microflora, presence of caries lesions, presence of visible plaque on the teeth, sugar consumption, presence of appliances in the mouth, difficulty in accessing oral and dental health care and sociodemographic and socioeconomic factors [1-4].

Saliva, which contains many different organic and inorganic substances, is an essential element that affects the oral cavity and tooth surfaces [5]. Changes in the amount and structure of saliva may cause a decrease in moisturizing, lubricating, remineralization, buffering, and antimicrobial effects. Thus, as the salivary pH (SpH) decreases, the risk of infection increases, and dental caries occur [6]. Saliva is considered the most critical host-related factor, which can shift the direction of dental caries activity to remineralization [7,8].

There is a strong connection between dental and overall health, and diet is an essential step in this regard [9,10]. Nutrition is crucial in developing and protecting oral and dental tissues, preventing diseases, and treating them when necessary [11,12]. Potential renal acid load (PRAL) determines the acid loads of foods or diets [13]. The decrease in the PRAL in the negative (-) direction indicates that the diet shifts to alkaline, while its increase in the positive (+) direction indicates that the diet shifts to acidity [14].

The Healthy Eating Index (HEI) is a diet quality index that measures compliance with the Diet Guidelines for Americans (DGA) and includes 13 dietary components [15]. HEI is essential in evaluating the relationship between diet quality and caries [16]. There has been limited research attention on the collective predictive role of salivary buffer capacity (SBC), PRAL, HEI, and dietary factors on dental caries. This study aimed to examine the prediction capability of SBC, PRAL, and HEI on children’s dental caries. The null hypothesis of the study was that dental caries cannot be predicted using SBC, PRAL, and HEI.

## Materials and methods

This is a cross-sectional study involving children who applied to Recep Tayyip Erdogan University, Faculty of Dentistry, Department of Pediatric Dentistry. The study was conducted between July and December 2021. Ethics committee approval was retrieved from Recep Tayyip Erdogan University, Faculty of Medicine, Ethics Committee (155/2021). The parents were informed in writing and verbally about the content, duration, and possible benefits of the study and were asked to sign the declaration of consent.

A simple random sampling method was preferred to represent the universe in the sample selection. In the literature review [16], the total sample size was found to be 138 using the G-Power software (Sydney, Australia) with a 0.3 (Cohen) effect size, 95% power, and 0.05 margin of error, based on the percentage measurement values of the methods. Thus, 150 patients were included in the study in order to prevent possible data loss.

Inclusion criteria

Patients aged 7-12 years, without any systemic disease or physical-mental disability, who agreed to answer the saliva collection procedure and the questionnaire and who consumed their meal and brushed their teeth at least two hours before the saliva collection process were included in the study.

Questionnaire (Hanna Instruments-HI-83141)

The language of the questionnaires was Turkish, and these were translated into English. The questionnaires had adequate reliability with Cronbach’s alpha coefficient (>0.70). Questions regarding demographic features were asked and recorded.

Dental examination

Before the examinations, the principal investigator (E.K) was initially trained and calibrated according to the WHO Basic Surveys Calibration Protocol for caries detection, coding findings, and recording. Firstly, the consistency of the decay, missing, filing, and teeth for primary teeth (dmft)/Decay, Missing, Filling, and Teeth for permanent teeth (DMFT) measurements of the investigator (E.K) via obtained data from the first 20 patients who was examined twice with an interval of two weeks. Concordance correlation analysis was used to estimate the intra-rater reliability and sufficient reliability was obtained (r=0.84). A dental examination was performed using a sterile dental Shepherd’s Hook explorer and mirror. Caries evaluation was performed by visual and digital panoramic radiographs. In cases in which caries detection was challenging, the bitewing radiography technique was used to support the panoramic radiograph. After the tooth was dried, each tooth was recorded as decayed (d), missing (m), or filled (f), and the burden of caries was determined according to the dmft/DMFT index. The patients were divided into two parts: subjects with low (dmft≤5, DMFT≤1) and high (dmft>5, DMFT>1) dental caries.

Collection of saliva samples and measurement of salivary buffer capacity

Approximately 1 mL of unstimulated saliva was collected using the Ericsson Method [17] in the next control session between 9-12 hours in the morning, in a calm environment, without any stress. As soon as saliva samples were taken, their buffering capacity was measured with a pH meter (Hanna Instruments-HI-83141). Since the measurement was made immediately, there was no need to store the saliva samples. The children spit into the phantom tubes, and after mixing the saliva with the calibrated tip of the pH meter device (Hanna Instruments-HI-83141), the pH was measured and recorded.

Dietary acidity

Macro- and micronutrient contents of foods (especially protein, phosphorus (P3-), potassium (K2+), magnesium (Mg2+), and calcium (Ca2+)) with a retrospective one-day nutritional record were obtained by using the BeBiS software (BeBiS 9, Turkey). The PRAL was calculated with these data. The formula arranged by Remer [14] according to the kidney acid load formed by the nutrients is as follows:

PRAL (mEq/day)=0.49 × protein (g/day) + 0.037 × phosphorus (mg/day) - 0.021 × potassium (mg/day) - 0.026 × magnesium (mg/day) - 0.013 × calcium (mg/day).

Healthy Eating Index

The foods' macro- and micronutrients were obtained using the BeBiS software (Ebispro for Windows, Stuttgart, Germany) with a retrospective one-day nutritional record. The HEI score was determined with the data obtained with this software. This index has some limitations as it analyzes only one day's nutrition record.

Statistical analysis

Statistical analysis was performed using Jamovi Software (Version: 2.3.21; Sydney, Australia). The relationship between demographic characteristics and caries indices was analyzed using the chi-square test. The distribution of data was tested with the Kolmogorov-Smirnov test. Because the data were normally distributed, the association of dental caries indices with PRAL, SBC, and HEI was analyzed using an independent sample t-test. A binomial logistic regression analysis was performed to predict the dental caries burden. The statistical significance level was adjusted to a=0.05.

## Results

A total of 150 children, 88 (58.6%) females and 62 (41.4%) males, were included in the study. No significant relationship was found between dmft/DMFT indices and demographic characteristics (Table 1).

**Table 1:** Association between dental caries and some demographic attributes dmft: decay, missing, filing, and teeth for primary teeth; DMFT: Decay, Missing, Filling, and Teeth for permanent teeth.

	dmft			DMFT			
	>5 (N=73)	≤5 (N=77)	p value	>1 (N=70)	≤1 (N=80)	p value	Total (N=150)
Gender			0.348			0.33	
Female	40.0 (54.8%)	48.0 (62.3%)		44.0 (62.9%)	44.0 (55.0%)		88.0 (58.7%)
Male	33.0 (45.2%)	29.0 (37.7%)		26.0 (37.1%)	36.0 (45.0%)		62.0 (41.3%)
Age range			0.463			0.181	
7-10	46.0 (63.0%)	44.0 (57.1%)		38.0 (54.3%)	52.0 (65.0%)		90.0 (60.0%)
11-12	27.0 (37.0%)	33.0 (42.9%)		32.0 (45.7%)	28.0 (35.0%)		60.0 (40.0%)
Socioeconomic status			0.337			0.109	
Low	23.0 (31.5%)	27.0 (35.1%)		25.0 (35.7%)	25.0 (31.2%)		50.0 (33.3%)
Mediocre	29.0 (39.7%)	22.0 (28.6%)		28.0 (40.0%)	23.0 (28.8%)		51.0 (34.0%)
High	21.0 (28.8%)	28.0 (36.4%)		17.0 (24.3%)	32.0 (40.0%)		49.0 (32.7%)
Toothbrushing habit			0.881			0.31	
Always	14.0 (19.2%)	14.0 (18.2%)		11.0 (15.7%)	17.0 (21.2%)		28.0 (18.7%)
Sometimes	55.0 (75.3%)	60.0 (77.9%)		54.0 (77.1%)	61.0 (76.2%)		115.0 (76.7%)
Never	4.0 (5.5%)	3.0 (3.9%)		5.0 (7.1%)	2.0 (2.5%)		7.0 (4.7%)

**Figure 1:**
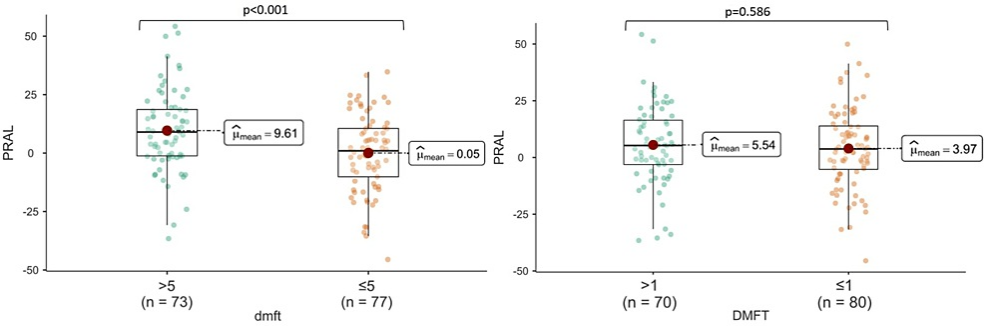
The relationship between PRAL and dental caries indices PRAL: potential renal acid load; dmft: decay, missing, filing, and teeth for primary teeth; DMFT: Decay, Missing, Filling, and Teeth for permanent teeth.

**Figure 2:**
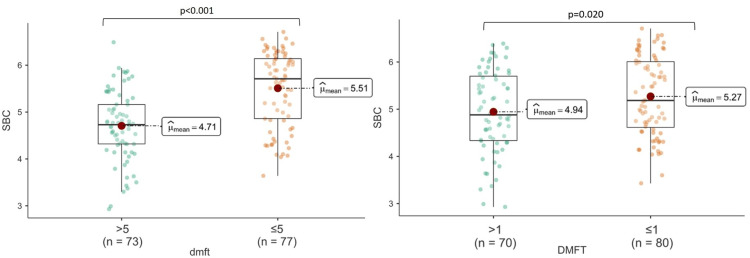
The relationship between SBC and dental caries indices SBC: salivary buffer capacity; dmft: decay, missing, filing, and teeth for primary teeth; DMFT: Decay, Missing, Filling, and Teeth for permanent teeth.

**Figure 3:**
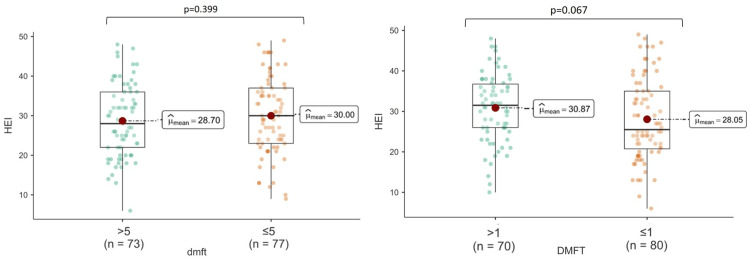
The relationship between HEI and dental caries indices HEI: Healthy Eating Index; dmft: decay, missing, filing, and teeth for primary teeth; DMFT: Decay, Missing, Filling, and Teeth for permanent teeth.

Significant differences were found between the low and high dental caries groups for dmft regarding PRAL and SBC (p<0.001); however, there was no significant difference regarding HEI (p>0.05). A significant difference was found between the low and high dental caries groups for DMFT in terms of SBC (p<0.05); however, there was no significant difference in terms of PRAL and HEI (Figures 1-3).

PRAL (OR=0.99, p=0.466), SBC (OR=3.5, p<0.001), and HEI (OR=1.02, p=0.331) were added to model 1, and the model was adjusted with gender, age range, socioeconomic features, and tooth brushing habit. The model explained 20% of the variance (McFadden’s R^2^=0.20) (Table 2).

**Table 2:** Binominal logistic regression analysis for dmft Adjusted with gender, age range, socioeconomic features, and tooth brushing habit. PRAL: potential renal acid load, SBC: salivary buffer capacity, HEI: Healthy Eating Index.

Predictor	Estimate	SE	Z	p	Odds ratio	95% CI Lower	95% CI Upper	McFadden’s R^2^
Model 1								0.20
Intercept	-6.76	1.6	-4.22		0	0	0.03	
SBC	1.25	0.27	4.59		3.5	2.05	5.97	
PRAL	-0.01	0.01	-0.73	0.466	0.99	0.97	1.02	
HEI	0.02	0.02	0.97	0.331	1.02	0.98	1.06	

PRAL (OR=1.01, p=0.373), SBC (OR=1.68, p=029), and HEI (OR=0.96, p=0.058) were added to model 2, and the model was adjusted with gender, age range, socioeconomic features, and tooth brushing habit. The model explained 8% of the variance (McFadden’s R^2^=0.08) (Table 3).

**Table 3:** Binominal logistic regression analysis for DMFT Adjusted with gender, age range, socioeconomic features, and tooth brushing habit. PRAL: potential renal acid load, SBC: salivary buffer capacity, HEI: Healthy Eating Index.

Predictor	Estimate	SE	Z	p	Odds ratio	95% CI Lower	95% CI Upper	McFadden’s R^2^
Model 2								0.08
Intercept	-1.5	1.38	-1.09	0.276	0.22	0.02	3.31	
SBC	0.52	0.24	2.19	0.029	1.68	1.06	2.68	
PRAL	0.01	0.01	0.89	0.373	1.01	0.99	1.03	
HEI	-0.04	0.02	-1.89	0.058	0.96	0.93	1	

**Figure 4:**
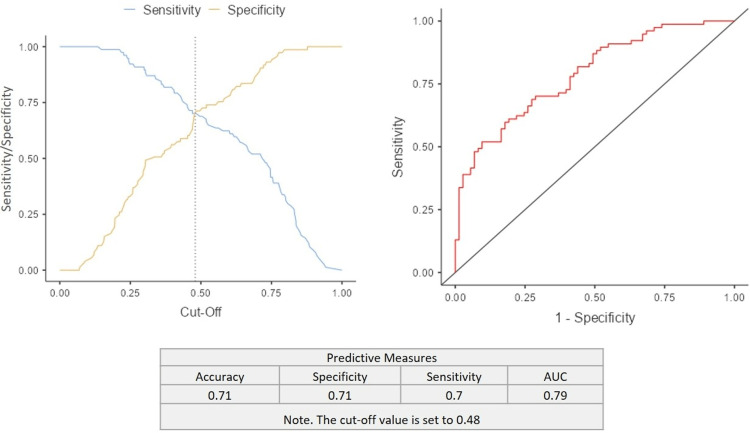
Cut-off plot and ROC curve presentation AUC: area under curve; ROC: receiving operating characteristic.

In the receiving operating characteristic (ROC) analysis, the optimal cut-off value of 0.48 for dmft was seen to have 70% sensitivity and 71% specificity. The cut-off plot and ROC curve are presented in Figure 4.

## Discussion

In our study, there was a significant relationship between SBC, PRAL, and caries in primary teeth. In the model we created using SBC, PRAL, and HEI, the strongest predictor was SBC. We can say that if we know SBC, we can make a significant guess whether the child has dental caries or not; at the same time, we can say that SBC affects dental caries much more than other factors.

The effectiveness of SBC depends on various factors such as systematic, genetic diseases, diet, hygiene habits, and medicines used [18-20]. Several studies have shown that salivary pH is a significant predictive factor for caries development [21-23], in agreement with the present study. In this study, SBC was the most effective predictor for dmft. The buffering capacity of saliva and fermentable carbohydrates affects the plaque's pH. When the saliva is unstimulated, the pH in the plaque is approximately between 6 and 7.3. The pH rises during the first five minutes after ingestion of most foods. Then, the pH drops to its lowest level, 6.1 or even less, approximately 15 minutes after food consumption. Plaque pH gradually returns to its resting pH unless there is an additional intake of fermentable carbohydrates. Demineralization occurs when acids diffuse through the plaque and pellicle into the liquid phase of the enamel between the enamel crystals. The resulting dissolution occurs at a pH of 5-5.5, the critical pH range for caries development. The dissolved minerals diffuse out of the tooth structure and into the saliva surrounding the tooth. The buffering capacity of saliva significantly affects the plaque's pH surrounding the enamel, thus preventing the progression of caries [7,8,24-26].

Acid-base balance affected by dietary habits is becoming increasingly noteworthy in medical fields. PRAL enables the estimation of endogenous acid production exceeding the alkaline level produced for specific amounts of food ingested daily [7]. Akyüz et al. [26] found that the PRAL value is positively correlated with grain and meat consumption and negatively correlated with fruit and vegetable consumption. Also, a negative correlation was exhibited between the number of caries and the consumption of milk and dairy products and between the number of fillings and fruit consumption. In our current work, a positive correlation was found with the number of primary tooth fillings. Also, it has been reported that the PRAL does not significantly correlate with the number of primary tooth caries, dmft, and dmft index scores.

Nowadays, foods with cariostatic and cariogenic properties are included in most meals. Therefore, the relationship between diet content and caries should be assessed. The negative correlation between HEI and dental caries has been shown in several studies [16,27]. However, the present study uncovered no association between HEI and dental caries, probably due to the presence of weak HEI (<50) in all children. The fact that the participants were from the same region and had similar socioeconomic conditions may have made it complicated to determine the relationship between HEI and dental caries. For a more accurate assessment, including children from different regions with different socioeconomic backgrounds may provide a more precise inference.

This study had some limitations; the generalizability was low because the involved population was in a limited region. Furthermore, dental caries has multifactorial etiology, and adding all the potential factors in the model is unfeasible. Also, each age has different deciduous and permanent teeth combinations; generalizing models for all ages may decrease predictive robustness.

## Conclusions

Within the limitations of the study, the established regression models have significantly predicted dental caries in deciduous teeth. SBC was the most influential factor in predicting dental caries compared to PRAL and HEI. There was a significant relationship between SBC, PRAL, and caries in deciduous teeth. In the model we created, the strongest predictor was SBC. However, the variance explanation capacity of dental caries in permanent teeth was not strong as in deciduous teeth.
